# On the Control of Acute Rodent Malaria Infections by Innate Immunity

**DOI:** 10.1371/journal.pone.0010444

**Published:** 2010-05-06

**Authors:** Beth F. Kochin, Andrew J. Yates, Jacobus C. de Roode, Rustom Antia

**Affiliations:** Department of Biology, Emory University, Atlanta, Georgia, United States of America; Singapore Immunology Network, Singapore

## Abstract

Does specific immunity, innate immunity or resource (red blood cell) limitation control the first peak of the blood-stage parasite in acute rodent malaria infections? Since mice deficient in specific immunity exhibit similar initial dynamics as wild-type mice it is generally viewed that the initial control of parasite is due to either limitation of resources (RBC) or innate immune responses. There are conflicting views on the roles of these two mechanisms as there is experimental evidence supporting both these hypotheses. While mathematical models based on RBC limitation are capable of describing the dynamics of primary infections, it was not clear whether a model incorporating the key features of innate immunity would be able to do the same. We examine the conditions under which a model incorporating parasite and innate immunity can describe data from acute *Plasmodium chabaudi* infections in mice. We find that innate immune response must decay slowly if the parasite density is to fall rather than equilibrate. Further, we show that within this framework the differences in the dynamics of two parasite strains are best ascribed to differences in susceptibility to innate immunity, rather than differences in the strains' growth rates or their propensity to elicit innate immunity. We suggest that further work is required to determine if innate immunity or resource limitation control acute malaria infections in mice.

## Introduction

Understanding what controls the initial decline in pathogen density during the acute phase of infections is an important and largely unsolved problem. Three mechanisms may cause this decline: (i) the specific immune responses of the host; (ii) the innate immune response of the host; and (iii) the availability of resources, such as target cells, that are required for pathogen replication.

During the acute phase of primary malaria infection the parasite grows exponentially to a high density through replication in red blood cells (RBCs), and subsequently declines. Different malaria strains reach different peak densities in this phase. The dynamics then become much more complex, and are strongly influenced by the interplay between specific immune responses and antigenic variation which allows the parasite to evade these specific responses [1]–[5].

Because of the wealth of data available on the early dynamics of parasite and RBCs in mice infected with *Plasmodium chabaudi*, we focus on this system. Specific immunity is unlikely to be controlling parasite growth during the acute phase of infection in this system. Mice lacking B or T cells exhibit early parasite dynamics that are very similar to those in wild type mice [6]–[9]. This observation leaves us with the resource limitation and innate immunity hypotheses.

Many theoretical studies have proposed that RBC limitation determines the acute parasite dynamics [10]–[15], but this viewpoint is supported by relatively little experimental data [16]. In this scenario the transient exhaustion of susceptible RBC causes the first decline in parasite numbers in the blood. Gupta et al. [10] introduced the possibility that RBC limitation played a major role during the initial stages of infection. McQueen and McKenzie [12] explored the consequences of allowing the parasite to infect only a limited subset of RBCs (of a specific age range). Antia et al. [14] and Mideo et al. [15] showed how the RBC limitation models could describe the dynamics of infection of mice with *P. chabaudi*. However, the ability of a model to describe the data does not make it correct.

In contrast, the predominant view in the experimental literature is that innate immunity is responsible for the initial control of infection [see, for example, [17]–[20]]. For example, mice lacking IFN-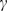 are not able to control the initial infection as well as wild type mice and often die [21], [22]. While a few pioneering theoretical studies explored how innate immunity may affect the dynamics of malaria infections [23]–[25], none of them has considered whether innate immunity can describe the early dynamics of infections with *Plasmodium chabaudi*. One major difficulty to overcome is our lack of a detailed quantitative understanding of the innate immune response. For example, the functional form of the innate immune response terms in the study by Dietz et al. [24] does not allow for a decline in parasitemia after the maximum parasitemia is reached. The study by Haydon et al. [23] does not include a decay term for the innate immune response, and so this alone would drive the parasite density to zero following the initial peak. The study by McQueen and McKenzie [25] considers how the incorporation of innate and specific immunity alters the dynamics observed in an RBC-limited model, but does not discuss whether innate immunity alone can explain the initial dynamics. This is the question that we address in this paper.

We consider a detailed dataset on the timecourse of infections of inbred mice with the AS and AJ strains of *Plasmodium chabaudi* [26]–[28]. In these experiments mice were infected with AS or AJ alone, or were co-infected with both. Co-infection was done in three ways: the strains were administered simultaneously, or one strain was given three days before the other. In single-strain infections, AJ reached higher densities and caused greater anemia than strain AS; in the co-infection experiments, AJ outcompeted AS. Previously, we showed that these data can be explained using a resource-limitation model [14] in which AJ is able to infect a larger proportion of red blood cells than AS. In this paper we determine if a model of parasite dynamics controlled by the innate immune response alone can also explain the data. We will also identify the factors underlying the different dynamics of the two strains in this (innate immunity) model.

## Methods

The model consists of equations for the density of parasite, 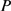, and the magnitude of the innate immune response, 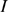. In accord with previous models, we let 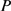 be the density of infected RBCs [3], [24], [29].

We focus on the role of innate immunity, rather than other factors such as RBC limitation, in the control of the parasite. Consequently, we assume the parasite grows exponentially (at rate 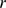) in the absence of innate immunity. This is predicted by the resource-limitation models if RBCs are in abundance and is a simple consequence of the repeated rounds of amplification of parasite number resulting from infection and bursting of RBCs. We let the magnitude of the innate immune response to the parasite, 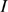, be the number of activated innate immune cells (*e.g.* phagocytic cells such as macrophages and dendritic cells). These cells produce inflammatory cytokines such as IFN-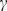, TNF-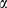 and IL-12 which have been shown to be upregulated following infection [21], [30]–[32].

A robust model of the innate immune response should include three key features which distinguish it from the adaptive (or antigen-specific) response [33]. First, innate immunity is dependent on the direct activation or recruitment of effector cells and consequently can be elicited more rapidly than the adaptive immune response, which involves cell proliferation by clonal expansion. Second, while recruitment is faster than replication, this limits the maximum magnitude of the innate response. Finally, innate immunity does not exhibit long-term memory – its magnitude decays in the absence of continued stimulation.

**Figure 1:**
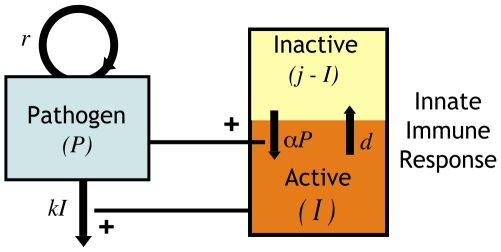
Schematic of model. In our model the density of the parasite, 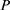, depends on two factors – its own replication (at rate 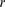) and its clearance by activated innate immune cells 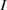 at rate 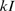. The total number of innate immune cells equals 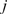 and they can be either in a resting or activated state. Since 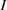 is the number of activated cells, the number of resting cells equals 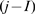. Resting innate immune cells are activated at rate 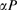, and revert back to the inactive state at exponential rate 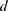.

We let the total number of cells of the innate immune system be constant at 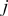, and the induction of an innate immune response is through the recruitment and/or activation of these cells rather than their proliferation or clonal expansion. The number of resting innate immune cells thus equals 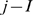. We use a mass-action term for the activation of these resting cells by exposure to the parasite, with rate constant 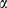. Activated innate immune cells clear infected RBCs at rate 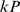 (by phagocytosis, reactive oxygen or other methods). Activated cells become inactivated at rate 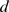. This model is shown schematically in Figure 1.
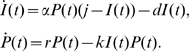
(1)This model can be extended to consider co-infections with two parasite strains, 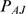 and 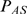 as follows:
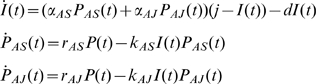
(2)


Without loss of generality we can scale the maximum level of innate immunity, 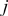 to unity. In our analysis we set the decay rate 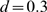 day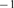, representing a half-life for the decay of the innate immune response in the absence of stimulus (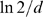) of approximately two days (see File S1, section 1). The remaining parameters (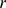, 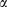 and 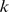) are estimated using the data from single infections with AS or AJ by minimizing the residual sum of squares. We then use these parameter values to predict the outcome of mixed infections as in Antia et al. [14].

In File S1, section 2, we show how the dynamics arising from this simple model of parasite growth and clearance in the blood are indistinguishable from those obtained with a fuller description of RBC infection and dynamics.

## Results

We compare the model predictions to data from experiments measuring parasite density of the AS and AJ strains of *P. chabaudi* following infection of C57BL/6 mice as described in the introduction.

**Figure 2:**
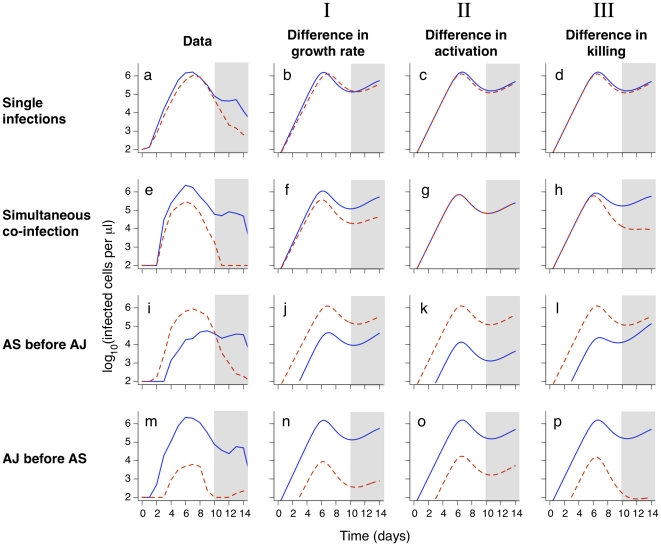
Experimental data and models for the dynamics of strains AS (red dashed lines) and AJ (blue solid lines). We use the model described in the text to investigate whether the dynamics of AS and AJ could be explained by (**I**) AJ having a higher growth rate than AS; (**II**) AJ inducing innate immunity more slowly than AS; or (**III**) AJ being less susceptible to killing by innate immunity than AS. Host Parameters: 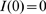 (that is, no innate immunity is activated at infection), 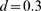 day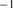 and 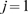. Strain parameters are estimated using the single infection data from days 2–10 only (unshaded area). Strain parameters, **I**: 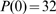 cells, 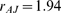 day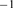, 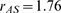 day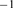, 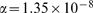 days
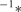
cells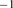, 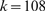 day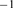. **II**: 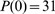 cells, 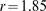 day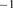, 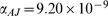 days
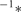
cells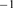, 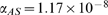 days
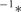
cells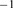, 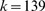. **III**: 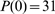 cells, 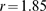 day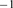, 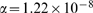 days
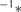
cells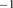, 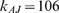 day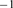, 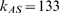 day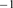. The left-hand column shows the mean parasite counts over time from experimental data for single infections (panel **a**; n = 11 (AJ) and n = 14 (AS)) and co-infections (panels **e, i, m**; n = 4, 4 and 5 respectively).

We begin with single infections (Figure 2, **a–d**). We see that the model (eqns. (1)) can describe the basic features of single infections – an initial exponential growth and subsequent control of the parasite. We then explore how differences in the model parameters for different strains can affect the peak parasite density, by allowing AS and AJ to differ in only a single parameter. Differences in peak parasite density between the two strains might be explained by differences in the following parameters of the model: (i) 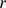, the growth rate; (ii) 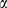, the rate constant for eliciting immunity; or (iii) 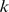, the rate of clearance by the innate immune response. We note that the parameter 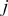, which describes the maximum magnitude of innate immunity (which may be thought of in this case to equal the total number of phagocytic cells) depends on the host and not the parasite strain. We find that AJ reaches a higher density than AS if it has a higher growth rate (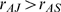: Figure 2, **b**), a lower rate of eliciting innate immunity (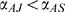: panel **c**), or a lower rate of being killed by innate immunity (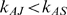: panel **d**). Fitting on days 2–10 after infection, we find that any one of these three possibilities is about equally good at explaining the single infection data (the residual sum of squares is very similar). After day 10, the observed parasite densities are lower then predicted by the model. This is not surprising since we do not include specific immunity which likely acts to control the parasite after day 10.

We then consider the competition between AJ and AS in hosts simultaneously infected with both strains (Figure 2, **e–h**). Using the parameters obtained from the single infection data we predict the outcome of simultaneous infections. We find that a difference in growth rate will allow the faster growing strain, AJ, to outcompete the slower growing parasite strain, AS (panel **f**). This is because the AJ strain will reach a higher density prior to both strains being controlled at the same rate by the innate immune response. We see a similar result following co-infection of a host when AJ and AS differ in their susceptibility to innate immunity (panel **h**). If, however, the strains differ in the rate at which they elicit innate immunity we get a different result. In this case the model predicts no competitive advantage for AJ if it simply activates the innate immune response at a slower rate then AS (panel **g**). This can be understood intuitively – in simultaneous infections the innate immune response clears both strains equally, irrespective of which strain elicits it most potently.

Little new insight is gained by looking at the model predictions for sequential infections. As in the case of co-infections the experimental results (panels **i, m**) show that AJ outcompetes AS (the ratio of AJ/AS increases, particularly when AS is present prior to AJ). This can be seen in simulations if the strains differ in either their growth rate or the rate they are killed by the innate immune response, but not if they differ only in the rates at which they activate innate immunity.

Caution is needed when interpreting parameter values. The parameters 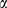 (activation rate) and 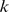 (killing rate) are degenerate as described in File S1, section 3. In other words multiple combinations of 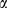 and 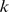 give the same dynamics.

In summary, our results indicate that control by innate immunity can explain the dynamics of AJ and AS in single and mixed infections. We note that a difference in the rate at which AJ and AS elicit immunity (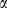) alone does not give rise to the observed competition dynamics in this model; there must also be a difference in either growth rate 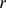 or susceptibility to innate immunity 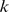. However, by estimating parasite growth rates using infected cell data from days 2, 3 and 4 only we found no significant difference in the growth rates of AS and AJ (mean 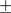 standard deviation: 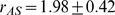 day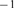, 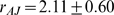 day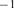; 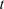-test, 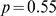), suggesting susceptibility to immunity is the most likely factor underlying strain differences in the innate immune control hypothesis.

## Discussion

This study explores one hypothesis for the control of the dynamics of acute malaria infections – that the innate immune system is responsible for the initial control of the parasite density in blood.

We have intentionally used a simple model. Much remains to be done to quantify innate immune responses experimentally, and there have been relatively few attempts to model them in detail. As a first approximation, we have modeled the immunostimulatory force as the number of infected cells and equated the magnitude of the innate immune response with the number of activated macrophages and other phagocytic cells. In this picture the activated phagocytes work by killing infected RBCs. Despite its simplicity, our model provides surprisingly close agreement with the qualitative features of the experimental data. As we obtain a better quantitative description of innate immune responses and independent estimates for the different parameters we hope to be able to make quantitative predictions.

We find that interaction between parasite growth and the innate immune system can indeed give rise to dynamics similar to those observed during acute infections of naive hosts. However this requires that the rate of decay of innate immunity is not too fast (we need a half life of days rather than hours). It is perhaps not very surprising that within certain parameter regions the model can generate the observed dynamics. Exponential growth followed by a contraction phase can be fitted by a multitude of models. Also, there are a large number of free parameters due to our lack of detailed understanding of the dynamics of the innate immune response and its interaction with the malaria parasite. This highlights the importance of obtaining independent estimates for as many of the parameters as possible. For example, if an independent estimate for the decay rate of innate immunity was very fast giving a half-life of less then one day, we could reject this model.

Our model allows parasite strains to differ in three ways – their initial growth rate, the rate at which they stimulate innate immunity and their susceptibility to innate immunity. We use our model to understand the differences between AS and AJ strains of *P. chabaudi*. Our results suggest that AJ, which reaches higher densities and outcompetes AS, either grows faster than or is more resistant to innate immunity than AS. The data give more support to the latter explanation.

In an earlier study Antia et al. [14] showed that a resource limitation model could explain the data described in this paper. Here we show that innate immunity can also reproduce the observed dynamics. We note that there are some minor aspects of the data that seem to be better described by one model or the other. When AS is given before AJ (Figure 2, panel i), the resource limitation model seems to better describe the switch from AS to AJ parasite dominance. However, we do not think this is significant enough to make one model more likely then the other. Indeed we feel that modeling alone may not be sufficient to convincingly discriminate between different biological hypotheses. The process of making models forces us to make explicit what are frequently vaguely formed assumptions regarding the underlying biology, and these assumptions can impact on model discrimination. Multiple variations are possible in the innate immunity framework presented here, while preserving the three key biological features of the model. For example, activation or killing rates might saturate as functions of pathogen or immune effector densities; and different arms of the innate immune response might be directed at infected RBCs and at free merozoites. Correspondingly, there are many plausible variations within the RBC limitation framework. When making RBC limitation models we are forced to ask how RBCs differ in their susceptibility to infection and their response following infection. We previously assumed that a single discrete age-range of RBCs was susceptible to infection [14]; some variations include continuously variable or even bimodal susceptibility of RBCs to infection as a function of age, or differences in the fecundity of infected RBCs with age.

There are two ways in which further studies could discriminate between the resource limitation and innate immunity models for *P. chabaudi* infections. One approach involves computing the relative levels of support for different models. In this case, where we do not know the exact forms of the terms of the resource limitation or innate immunity this approach would involve comparing families of different innate immunity and resource limitation models. The advantage of this approach is that it could be undertaken with the existing data. The main problem with this approach is that while we have a reasonable quantitative biological understanding of resource limitation we have a much poorer basic quantitative understanding of innate immunity. We are not certain to have, even in the broader family of models, sufficiently accurate terms for innate immunity.

The alternative is further experimental studies to tease out more cleanly the relative contributions of resource limitation or innate immunity. In this view, further progress is most likely with experiments that test predictions that are independent of the details of the underlying models. Models, by forcing rigorous thought and explicit consideration of the assumptions behind verbal arguments might aid the design of key experiments.

The data used in this study comes from C57Bl/6 mice infected with two strains of P. chabaudi. However, provided the mice survive the acute phase on infection, qualitatively similar early dynamics (exponential growth followed by a decline) are observed following infections with different strains of parasite (such as P. berghei, and non-lethal P. yoelli) and host (such as BALB/c and C57Bl/10) [31], [34], [35].

We note that we do not consider adaptive immune responses. For the two strains of a rodent malaria examined here, the contribution of adaptive immune responses only becomes significant well after the acute parasitemia has peaked [6]–[9]. The timescales of infection in human malaria are different and specific immunity may well contribute to the control of the first peak in parasitemia [25], [36]. The *P.chabaudi* model system does however give us a wealth of experimental data which may help us develop hypotheses for the regulation of potentially more complex human malaria infections.

## Supporting Information

File S1 Appendix.
(0.26 MB PDF)
