# Supplementary material for: On the Control of Acute Rodent Malaria Infections by Innate Immunity
Source: PLoS One. 2010 May 6;5(5):e10444. doi: 10.1371/journal.pone.0010444 (PMC2865546; doi:10.1371/journal.pone.0010444)
Supplement: File S1 — Appendix. (0.26 MB PDF) [file pone.0010444.s001.pdf]

Appendix

1. Innate immunity decay rate

In this paper we use a decay rate of 0.3 days^{-1}. This value for d represents a biologically reasonable guess – we were unable to get an independent estimate for the decay rate as very little work has been done to quantify innate immunity.

We note that agreement between the model and the data requires that the half-life of the innate immune response be on the order of days, rather then hours – else we do not get a decline in parasite density following the initial maximum in parasitemia. When innate immunity decays more rapidly, the parasite density simply increases monotonically to a plateau. Figure S1 shows the model output for a single infection with the decay rate of innate immunity varying between 2 days^{-1}(half-life of about 8 hours) and 0.1 days^{-1}(half-life of one week). Changing the value of d slightly (as long it remains less then about 0.7) would not change any of our conclusions.

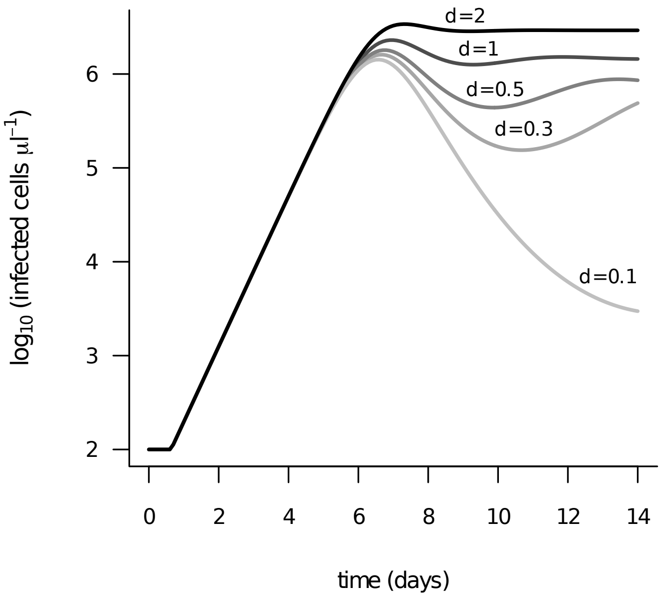

Figure S1: Varying the decay rate for innate immunity Parameters as for strain AJ in figure 2 column III. $I(0) = 0$, $P(0) = 31$ cells, $J = 1$, $r = 1.85$ days^{-1}, $\alpha = 1.22 \times 10^{-8}$ days^{-1}∗cells^{-1}, $k = 106$ days^{-1}. The decay rate for innate immunity, d, varies between 2 days^{-1} (top line) and 0.1 days^{-1} (bottom line).

We did not fit d because the best fit was obtained with d equal to zero, which is biologically unreasonable as we a key characteristic of innate immunity is that it decays rapidly. The low estimate of d might arise in part because of a small contribution of specific immunity which we expect to gradually come into play around day 10.

2. Model with RBC dynamics

In the text we present a simple ordinary differential equation (ODE) model for the control of malaria in the blood by innate immunity. This model includes equations for the infected red blood cells (RBC) and innate immunity only. Here we show that a more biologically detailed model that follows the dynamics of the entire RBC population produces the same results as the simple ODE model, as long as RBCs are not limiting.

We take the model developed in Antia et al. [1] describing the dynamics of free merozoites and of infected and uninfected RBCs, and incorporate killing by the innate immune response. This model explicitly incorporates the well-defined lifetime of RBCs in the blood; the time delay between infection of a red blood cell and its bursting to produce new merozoites; and the delayed response of the haematopoetic system to anemia induced by infection. As in the model presented in the main text, an immune response is generated in response to infected RBCs and results in the removal of infected RBCs through a simple mass-action killing term.

The model is described below. Here $x(t,\tau)$ is the population density of uninfected RBCs of age τ (= time since release into blood) at time t; $y(t,\tau,s)$ is the population density RBCs infected at age τ, at a time s since infection, at time t; $Z(t)$ is the density of free merozoites in the blood; $X(t)$ is the total number of uninfected RBCs in the blood; $Y(t)$ is the total number of infected RBCs in the blood. All populations are measured in units of cells or merozoites per microlitre. $I(t)$ measures the activated innate immune response.

$$\frac{\partial x}{\partial t}+\frac{\partial x}{\partial \tau} = -\beta x(t,\tau)Z(t) \tag{1}$$

$$X(t) = \int_0^{\tau_{RBC}} x(t,\tau)\mathrm{d}\tau \tag{2}$$

$$x(t,0) = F_0\frac{\theta^\kappa}{\theta^\kappa+(X(t-T))^\kappa} \tag{3}$$

$$\frac{\partial y}{\partial t}+\frac{\partial y}{\partial \tau}+\frac{\partial y}{\partial s} = -kI(t)y(t,\tau,s) \tag{4}$$

$$y(t,\tau,s=0) = \beta x(t,\tau)Z(t) \tag{5}$$

$$Y(t) = \int_0^{t_m}\mathrm{d}s\int_0^{\tau_{RBC}}\mathrm{d}\tau\; y(t,\tau,s) \tag{6}$$

$$\begin{aligned}\frac{\mathrm{d}Z(t)}{\mathrm{d}t} &= -d_m Z(t) + m\int_{\tau=0}^{\tau_{RBC}} y(t,\tau,s=t_m)\mathrm{d}\tau \\ &\quad -Z(t)\int_{\tau=0}^{\tau_{RBC}}\int_{s=0}^{t_m}\beta(x(t,\tau)+\lambda y(t,\tau,s))\mathrm{d}\tau\mathrm{d}s \end{aligned}\tag{7}$$

$$\frac{\mathrm{d}I(t)}{\mathrm{d}t} = \alpha Y(t)(J-I(t)) - d_i I(t) \tag{8}$$

In addition to the inclusion of innate immunity, we have made two changes to the original resource-limitation model. First, we allow all RBCs to be susceptible to infection, rather than restrict infection to particular age ranges of RBCs ($\beta=\beta(\tau)$). Second, we allow for a differential ability of merozoites to enter uninfected and infected RBCs; infected cells are a factor λ less susceptible to re-infection than uninfected cells ($0\le\lambda\le 1$). In the original model, both cell types absorbed free merozoites equally efficiently ($\lambda=1$). In any case, we assume that merozoites entering infected RBCs are simply lost.

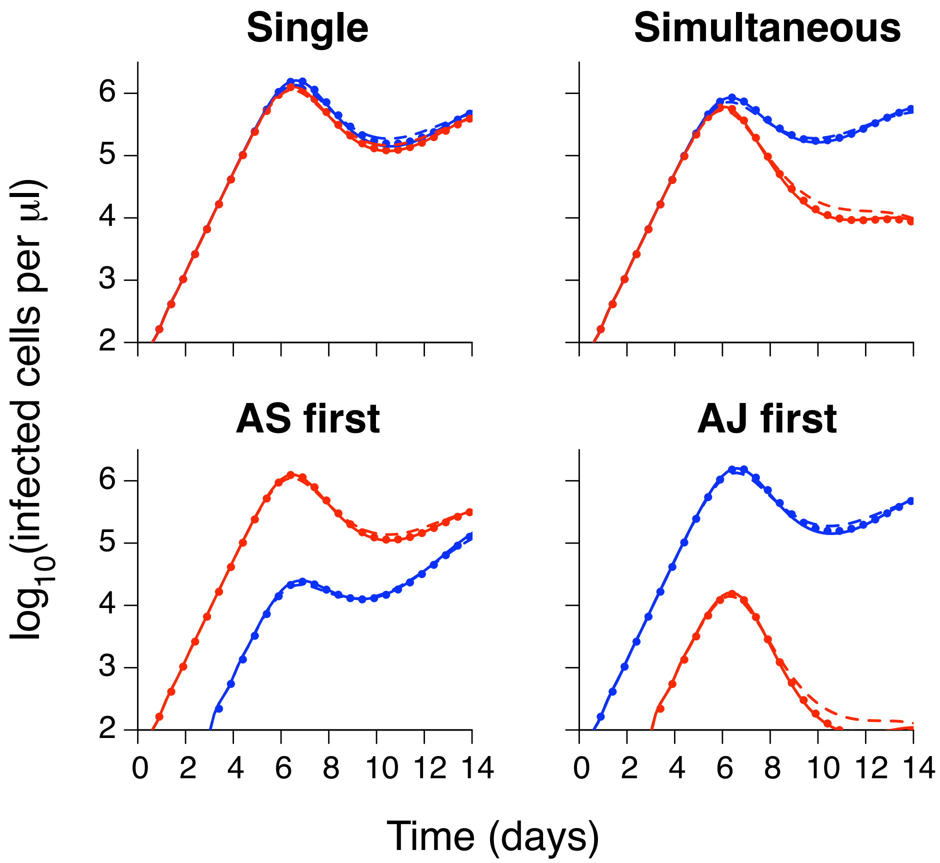

Figure S2: Models with and without total RBC for the dynamics of strains AS (red) and AJ (blue) Strain parameters as in figure 2 column III. $I(0) = 0$, $d_i = 0.3$ days^{-1} and $J = 1$, $m = e^r = e^{1.85}$, $\alpha = 1.22 \times 10^{-8}$ days^{-1}∗cells^{-1}, $k_{AJ} = 106$ days^{-1}, $k_{AS} = 133$ days^{-1}. For simple model: $P(0) = 31$ cells, $P(3) = 110$ cells (starting value for infections introduced on day 3). For full model: $Y(0) = 25$ cells, $Y(3) = 90$ cells. For RBC parameters see Antia 2008. solid line: full model, $\lambda = 0$; dashed line: full model, $\lambda = 1$; points = simple model.

As in Antia et al. [1], to generate simulations we convert this PDE model into sets of coupled ODEs. We break the age ranges of healthy RBCs into 40 compartments and infected RBCs into 20 compartments, and use 20 transit compartments to simulate the RBC production delay.

As shown in Figure S2 this model can produce the same infected cell dynamics as the simple ODE model presented in the main text. The growth rate r in the main text is related to the parameters of this larger model through $\ln m = rt_m$. Closest agreement between the two models is obtained in the limit $\lambda = 0$, when merozoites are assumed not to be able to enter infected red blood cells. Allowing for RBC superinfection ($\lambda = 1$) results in infected RBC being a 'sink' for the free parasite, an effect that increases with infected cell numbers and so progressively slows parasite growth.

3. Degeneracy

The simple innate immunity ODE model has one observed variable P, the number of infected cells, and one unobserved variable I, the magnitude of the activated innate immune response. Not only is I unobserved, but we also have no prior information on either α (activation rate) or k (killing rate). The parameters α and k are degenerate due to this lack of data. We can remove either of these parameters from the single strain model by rescaling P or I respectively. In the multi-strain model we can remove one of α_{AJ},α_{AS},k_{AJ} or k_{AS}.

To remove k from the single infection model we rescale innate immunity by letting $F(t) = kI(t)$. We then have:

$$\begin{aligned} \dot{F}(t) &= \alpha P(t)(\hat{j} - F(t)) - dF(t), \\ \dot{P}(t) &= rP(t) - F(t)P(t). \end{aligned} \tag{9}$$

where $\hat{j} = jk$.

We can remove k_{AJ} from the two-strain model by letting $F(t) = k_{AJ}I(t)$. The model then becomes:

$$\begin{aligned} \dot{F}(t) &= (\alpha_{AS}P_{AS}(t) + \alpha_{AJ}P_{AJ}(t))(\tilde{j} - F(t)) - dF(t) \\ \dot{P_{AS}}(t) &= r_{AS}P(t) - \tilde{k}F(t)P_{AS}(t) \\ \dot{P_{AJ}}(t) &= r_{AJ}P(t) - F(t)P_{AJ}(t) \end{aligned} \tag{10}$$

with $\tilde{j} = jk_{AJ}$ and $\tilde{k} = k_{AS}/k_{AJ}$.

References

1. Antia R, Yates A, de Roode JC (2008) The dynamics of acute malaria infections. I. Effect of the parasite's red blood cell preference. Proc Biol Sci 275: 1449–1458.
